# De Novo Transcriptome Sequencing Analysis Revealed the Expression Patterns of Genes in Different Organs and the Molecular Basis of Polysaccharide Synthesis in *Bletilla striata*

**DOI:** 10.3390/genes16050558

**Published:** 2025-05-06

**Authors:** Wenkui Liu, Jinxing Jiang, Zhonghai Tang, Zemao Yang, Jingping Qin

**Affiliations:** 1 College of Bioscience and Biotechnology, Hunan Agricultural University, Changsha 410128, China; liuwenkui0320@163.com (W.L.); 19573191769@163.com (J.J.); 2 College of Food Science and Technology, Hunan Agricultural University, Changsha 410128, China; tangzh@hunau.edu.cn; 3 Institute of Bast Fiber Crops, Chinese Academy of Agricultural Sciences, Changsha 410205, China; yangzemao@caas.cn

**Keywords:** *Bletilla striata*, transcriptome, different organs, polysaccharide synthesis

## Abstract

Background: *Bletilla striata* (Thunb.) Rchb.f., a perennial medicinal plant in the genus Bletilla of the Orchidaceae family, is renowned for its hemostatic, anti-inflammatory, and tissue-regenerative properties. Despite the established importance of polysaccharides as key bioactive components in *B. striata*, the genes and molecular mechanisms underlying their synthesis remain largely unexplored. Methods: This study conducted transcriptomic analysis on the roots, tubers, and leaves of *B. striata*, and identified gene expression profiles and candidate genes for polysaccharide synthesis in different organs. Results: The results indicated that there were 7699 differentially expressed genes (DEGs) in Tuber vs. Leaf, 7695 DEGs in Luber vs. Root, and 6151 DEGs in Tuber vs. Root. There were significant differences in polysaccharide metabolism pathways (photosynthesis, starch, and sucrose metabolism) in different organs of *B. striata*. The overall enrichment levels were ranked as tubers > leaves > roots. It is worth noting that enzyme genes involved in polysaccharide synthesis exhibit significant organ specificity, with *HK* genes expression significantly higher in roots than in tubers and leaves, *PMM*, *GMPP*, *pgm*, and *UGP2* genes highly expressed in tubers, while *scrK, manA*, and *GPI* genes have similar expression levels in the three organs. Conclusions: These findings identify key enzyme genes involved in the synthesis of polysaccharides in *B. striata*, providing a theoretical framework for enhancing its medicinal value through genetic improvement.

## 1. Introduction

*Bletilla striata* (Thunb.) Rchb.f., belonging to the family Orchidaceae, is an important traditional Chinese medicinal (TCM) plant. It is characterized by a bitter, sweet, astringent, and slightly cold taste with therapeutic properties understood to have an affinity for the lung, liver, and stomach meridians [1]. *B. striata* was first documented in the historical text ‘Divine Husbandman’s Classic’ [2], where its tubers were noted for their common medicinal use as an astringent, hemostatic, anti-inflammatory, and muscle-regenerating agent [3]. Currently, research on *B. striata* primarily focuses on the isolation of chemical components and exploring their pharmacological functions. The main constituents that have been isolated from *B. striata* include polysaccharides, biphenyls, phenanthrenes, flavonoids, triterpenoids, phenols, steroids, and other compounds [4].

*B. striata* polysaccharide (BSP) is a key bioactive ingredient in the plant and consists of glucose and mannose [5]. Research indicates that purified polysaccharides from *B. striata* can serve as skin-protective agents with strong antioxidant capabilities and anti-melanogenesis properties [6]. Additionally, these polysaccharides have potential applications in the prevention of Alzheimer’s disease, amelioration of morphological damage in the hippocampus and cortex, and reduction in the expression of β-secretase proteins [7]. BSP has gained significant traction in biomedical applications owing to its exceptional biocompatibility, cost-effective raw material sourcing, and versatile processability into diverse formulations, including hydrogel, microspheres, microparticles, microneedles, and sponges [8,9,10,11,12,13,14]. For example, BSP-prepared hydrogels can be effectively used as wound dressings because of their excellent wound-healing properties [15]. While *BSP* offers numerous benefits, the scarcity of genetic data impedes a comprehensive understanding of the biosynthesis mechanism of BSP.

In *B. striata,* the primary medicinal tissue is the tuber, while its roots and leaves are often discarded. However, some researchers have demonstrated that the volatile extracts from the roots, buds, and above-ground parts of *B. striata* have potent anti-cancer properties. These volatile constituents also exhibit a degree of tissue specificity [16]. Additionally, two new stilbenes, namely 5-methoxy-6-(4-methoxystyryl) benzofuran (1) and 6-(4-methoxystyryl) benzofuran-5-ol (2), have been identified in the leaves and stems of *B. striata*. These compounds have demonstrated potential anti-SARS-CoV-2 activity [17]. Therefore, the evidence from recent research suggests that aside from the tubers, the rest of the organs of *B. striata* also have potential medicinal value. An in-depth study of the differences between the plant’s various organs is of great significance for the further development and utilization of its resources. While differences in BSP accumulation have been reported in the roots, tubers, and leaves, the precise synthesis mechanism of this polysaccharide is still not well understood.

The accumulation of polysaccharides is commonly modulated by enzymes and regulatory factors. Tissue-specific transcriptomics is an effective approach to unravel the regulatory mechanisms of active ingredient biosynthesis [18]. For instance, through sequencing of the full-length transcriptome from leucocephala suspension cells, 23 4-coumarate-CoA ligase (4CL) genes were identified. *Bs4CL* plays a significant role in various metabolic processes, including lipid metabolism, α-linoleic acid metabolism, and the production of other secondary metabolites. The expression pattern of these *Bs4CL* genes is tissue-specific in different tissues [19]. The sugars will eventually be exported transporter (SWEET) family is a well-known bidirectional transporter. They facilitate sugar translocation between source and reservoir tissues in plants [20]. In *B. striata*, 23 *BsSWEET* genes have been identified, exhibiting tissue-specific expression patterns. Among these, *BsSWEET1*, *BsSWEET2b*, and *BsSWEET3a* are most strongly expressed in tubers, leaves, and roots, respectively [21].

Omics techniques have partially clarified BSP’s biosynthetic pathway. However, its transcriptional regulatory network and the regulatory mechanisms of key rate-limiting enzymes are still unknown [22]. A MYB transcription factor in *B. striata* has been found to regulate eight key BSP biosynthesis-related genes, including *sacA1*, *HK1*, *scrK1*, *scrK2*, *GPI1*, *manA1*, *GMPP1*, and *UGP2_1*. Despite these findings, their specific role in the BSP synthesis process is not clear [23]. The polysaccharide content in tubers of *B. striata* varied significantly, depending on the variety and the year of cultivation [24]. However, most of the studies focus on biennials or triennials, with fewer studies investigating younger *B. striata* plants, which may be masking valuable information about the biosynthesis pathway of the active ingredient in the early stages.

This study employed transcriptome sequencing analysis to explore the tissue-specific genes in different tissues of *B. striata*, identify the candidate enzyme genes involved in BSP biosynthesis, and determine the expression patterns of these genes in different organs. The results of this study provide a basis for analyzing the polysaccharides synthesis mechanism in *B. striata* and provide a reference for the resource utilization of this medicinal plant.

## 2. Materials and Methods

### 2.1. Plant Materials and RNA Extraction

All of the *B. striata* plant material used in this study was cultivated at Hunan Agricultural University. Seed Pods were collected from Cili County, Zhangjiajie City, Hunan Province. The pods were surface sterilized with 75% ethanol and washed three times with ddH_2_O. The seeds were sown on Murashige and Skoog (MS) solid plates containing 30 g/L sucrose at 25 ± 1 °C under a 16 h light/8 h dark photoperiod. Roots, tubers, and leaves from 5-month-old seedlings were sampled for RNA extraction. The samples collected were immediately frozen in liquid nitrogen and stored at −80 °C. Total RNA was extracted using the RNA kit (15NT) (Agilent Technologies, Santa Clara, CA, USA). RNA purity and integrity were monitored by a NanoDrop 2000 spectrophotometer (NanoDrop Technologies, Wilmington, DE, USA) and a Bioanalyzer 2100 system (Agilent Technologies, CA, USA). RNA contamination was checked by 1.5% agarose gel electrophoresis.

### 2.2. Library Construction and Sequencing

To isolate mRNA, magnetic beads coated with Oligo (dT) were utilized. The collected mRNA was subsequently broken down into smaller segments via a fragmentation buffer at an appropriate temperature. The process began with random hexamer-primed reverse transcription to generate first-strand cDNA, after which, second-strand cDNA was synthesized. The cDNA was then purified using AMPure XP Beads (Beckman Coulter, Brea, CA, USA). The cDNA fragments obtained from previous steps were amplified by PCR, and the products were purified via Ampure XP Bead to obtain the final library. Subsequently, the DNBSEQ-T7 high-throughput sequencing platform was utilized for the sequencing of various samples.

### 2.3. De Novo Assembly and Annotation of Gene Functions

SOAPnuke (V2.1.0) [25] was employed to filter the sequencing data through the following steps: (1) reads containing sequencing joints were eliminated; (2) reads with an N ratio exceeding 0.5% were discarded; (3) reads were considered low-quality and subsequently discarded if bases with a Qphred score ≤ 20 accounting over 50% of the entire read length. Clean reads are obtained after filtering. The reference transcriptome was assembled using all of the samples’ clean reads by Trinity (V2.10.0) [25] software. Then, all of the functional annotations were performed by blasting the Non-Redundant Protein Sequence Database (NR), Gene Ontology (GO), Kyoto Encyclo-pedia of Genes and Genomes (KEGG), Eukaryotic Orthologous Groups (KOG), and SwissProt Database (Swiss-Prot) databases using Diamond Blastx (V0.9.24).

### 2.4. Differentially Expressed Genes (DEGs) Analysis

For each sample, the clean data were aligned with the assembled reference transcriptome. Gene read counts were then obtained from the mapping process using Bowtie2 (V2.3.5) [26]. Fragments Per Kilobase Per Million bases (FPKM) were used to estimate gene expression levels using RSEM (V1.3.0) [27]. DEGs were identified using DESeq2 (V1.22.2) [28] with the threshold: FDR (false discovery rate) < 0.05, |log2FC (fold change)| > 1. DEGs were subjected to GO and KEGG functional enrichment. Hypergeometric distribution was used for GO enrichment analysis, and the GO term with Q-value ≤ 0.05 was selected as the significantly enriched GO entry. KEGG pathway analyses were conducted using KOBAS (V3.0) [29]. Pathways with a Q-value ≤ 0.05 were identified as statistically significant in terms of enrichment in DEGs. For data visualization, a heatmap and plots of GO and KEGG enrichment pathways were generated using an online data analysis and visualization platform (https://www.bioinformatics.com.cn, accessed on 13 August 2024).

### 2.5. Quantitative Reverse Transcriptase Polymerase Chain Reaction (qRT-PCR) Analysis

The expression patterns of enzyme genes involved in polysaccharides biosynthesis in roots, tubers, and leaves of *B. striata* were investigated by qRT-PCR. Reverse transcribed cDNA served as the template for qRT-PCR, which was carried out using a SYBR Green PCR Kit (Vazyme, Nanjing, China) on a Light Cycler 480 system (Roche, Rotkreuz, Switzerland). The specific primer sequences are presented in Table S1. The relative mRNA expression levels were determined using the 2^−ΔΔCT^ method [30].

### 2.6. Statistical Analysis

Principal Component Analysis (PCA) was performed based on FPKM for all samples. Statistical analysis was conducted using SPSS 25.0 software. One-way ANOVA with Tukey’s post hoc test was employed to assess group differences. Significant differences between groups (*p* < 0.05) are denoted by distinct letters.

## 3. Results

### 3.1. Sequencing and Transcriptome Assembly

**Figure 1:**
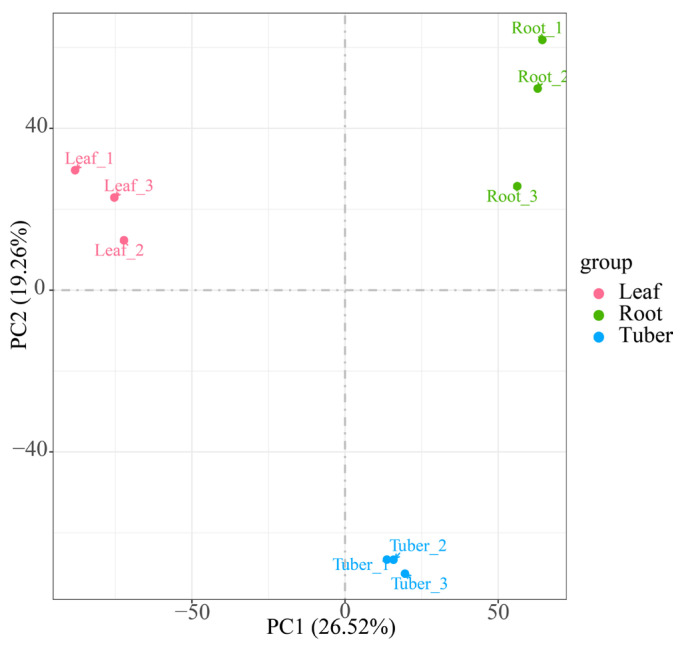
PCA plot illustrating the transcriptome profiles of *B. striata* sample based on FPKM (PC1: 26.52%, PC2: 19.26%).

To elucidate organ-specific gene expression patterns in *B. striata*, we performed transcriptome sequencing on root, leaf, and tuber samples. Firstly, raw reads were processed to remove low-quality reads, resulting in the acquisition of 55,981,637,400 bp of high-quality clean reads (Table S2). In the filtered data, 96.74% of the bases exceeded the quality score with Q30, while the GC content ranged from 43.84% to 46.05%. Principal Component Analysis (PCA) analysis showed that Principal Component (PC) 1 can explain 26.52% of the overall variation in the data, making it the largest source of variation, and PC2 can explain 19.26% of the overall variation in the data (Figure 1). Three clusters were formed according to the organization of different samples. The aforementioned results demonstrated that the sequencing data were of high quality and suitable for subsequent analysis. A total of 175,440 unigenes were generated from the transcriptome assembly. Among them, 66,031 (37.64%) transcripts were less than 500 bp, 35,588 (20.28%) ranged from 500 to 1000 bp, 36,767 (20.96%) were between 1 and 2 kb, and 37,054 (21.12%) exceeded 2 kb (Table S3).

### 3.2. Gene Functional Annotation

**Figure 2:**
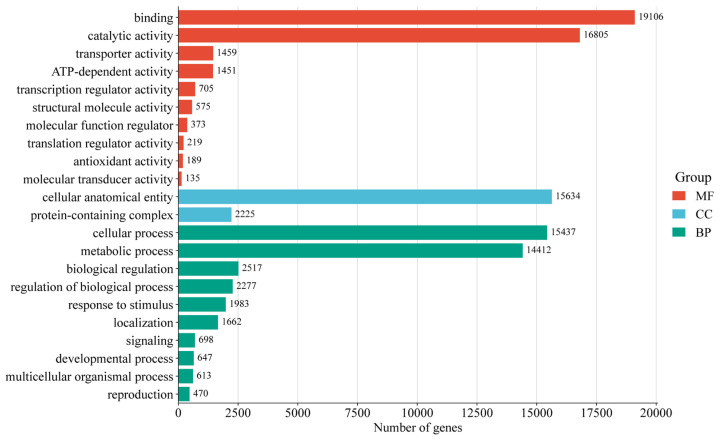
GO categories of all unigenes.

**Figure 3:**
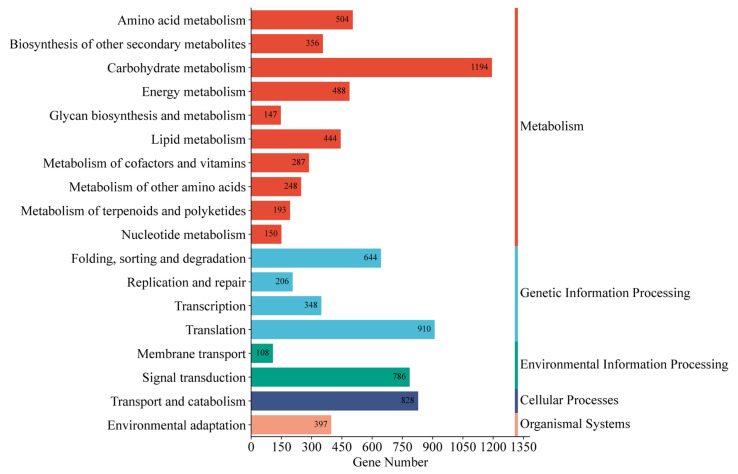
KEGG metabolism pathway categories of all unigenes.

Functional annotations were assigned to 96,418 unigenes (56.85% of total) by comparing them with five databases (Table S4). The functional annotation analysis revealed that a majority of unigenes (57,530; 59.67%) were aligned with the SwissProt database, followed by NR (38,542; 39.97%). GO classification successfully annotated 30,554 (31.69%) unigenes into three major GO categories: biological processes (BP), cellular components (CC), and molecular functions (MF) (Figure 2). Within the biological processes category, cellular processes (15,437 unigenes) and metabolic processes (14,412 unigenes) were the most enriched terms. Under cellular components, cellular anatomical entities (15,634 unigenes) and protein-containing complexes (2225 unigenes) were the most enriched terms. In the category of molecular functions, binding (19,106 unigenes) and catalytic activities (16,805 unigenes) were the most enriched terms. Additionally, 12,848 (13.33%) unigenes were annotated through the KEGG (Figure 3). The unigenes were classified into five categories, including cellular processes, environmental information processing, genetic information processing, metabolism, and organismal systems. For all KEGG categories sorted by the number of unigenes, the top pathway was carbohydrate metabolism (1194 unigenes), followed by translation (910 unigenes) of genetic information processing and transport, and catabolism (828 unigenes) of cellular processes. The metabolism pathway consisted of 18 KEGG categories, including signal transduction (786 unigenes), folding, sorting, and degradation (644 unigenes), amino acid metabolism (504 unigenes), and environmental adaptation (397 unigenes), and other pathways demonstrated progressively lower representation.

### 3.3. Analysis of DEGs in Different Organs

**Figure 4:**
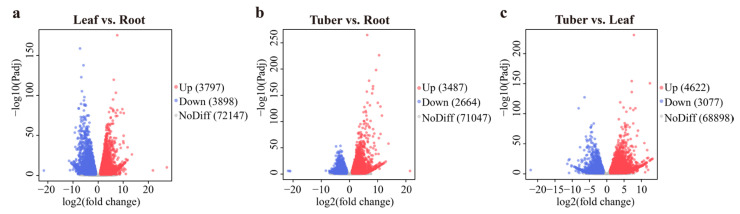
Differentially expressed genes (DEGs) analysis of *B. striata* in various organs: (**a**) the DEGs in the Leaf vs. Root group; (**b**) the DEGs in the Tuber vs. Root group; (**c**) the DEGs in the Tuber vs. Leaf group.

DEGs were identified from the roots, tubers, and leaves of *B. striata* (Figure 4). The most DEGs were found in the Tuber vs. Leaf group, with 4622 up-regulated and 3077 down-regulated genes. In contrast, the Leaf vs. Root group exhibited 3897 up-regulated and 3898 down-regulated genes. The Tuber vs. Root group had the lowest number of DEGs, with 3487 genes up-regulated and 2864 genes down-regulated.

**Figure 5:**
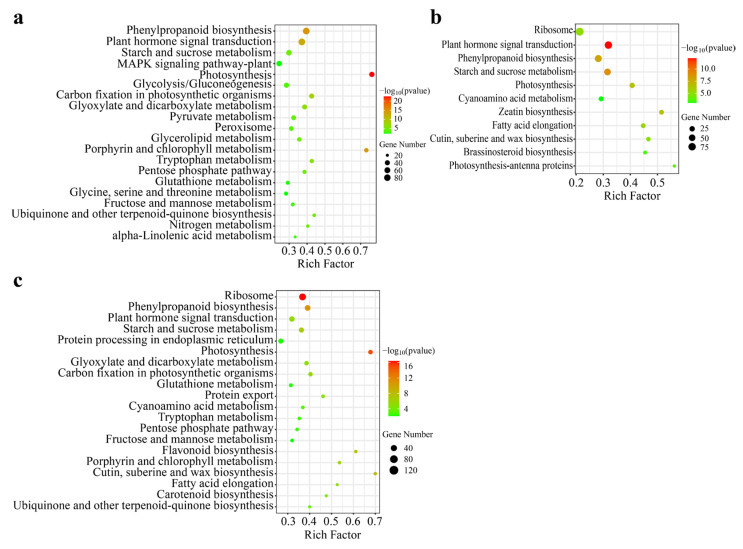
KEGG pathways enrichment analysis of the DEGs in Leaf vs. Root, Tuber vs. Root and Tuber vs. Leaf groups. Panel (**a**) DEGs in the Leaf vs. Root group. (**b**) DEGs in Tuber vs. Root group. (**c**) DEGs in Tuber vs. Leaf group.

To elucidate the functional roles of DEGs of *B. striata* in different organs, we performed a KEGG enrichment analysis. The KEGG pathway analysis revealed significant enrichment of 33, 11, and 25 pathways in Leaf vs. Root, Tuber vs. Root, and Tuber vs. Leaf, respectively. The top 20 enriched pathways are presented in Figure 5. Among the metabolic pathways analyzed, photosynthesis was identified as the most significantly enriched pathway. Other metabolic pathways included starch and sucrose metabolism, and fructose and mannose metabolism. These pathways were particularly significant in Leaf vs. Root (Figure 5a). The Tuber vs. Root comparison showed plant hormone signal transduction as the most significant pathway, with photosynthesis and starch and sucrose metabolism also demonstrating significant enrichment (Figure 5b). Similarly, in the Tuber vs. Leaf comparison, photosynthesis was identified as the most prominent pathway (Figure 5c). Notably, polysaccharide synthesis-related pathways observed in Leaf vs. Root also exist in Tuber vs. Leaf, such as starch and sucrose metabolism, fructose and mannose metabolism. Comparative analysis of DEGs associated with these significant pathways revealed significant differences in pathways such as photosynthesis, starch, and sucrose metabolism across three organs. This suggests that the organ-specific distribution of polysaccharide metabolism pathways leads to differences in polysaccharide accumulation.

### 3.4. Identification of Genes Involved in Polysaccharides Biosynthesis

**Figure 6:**
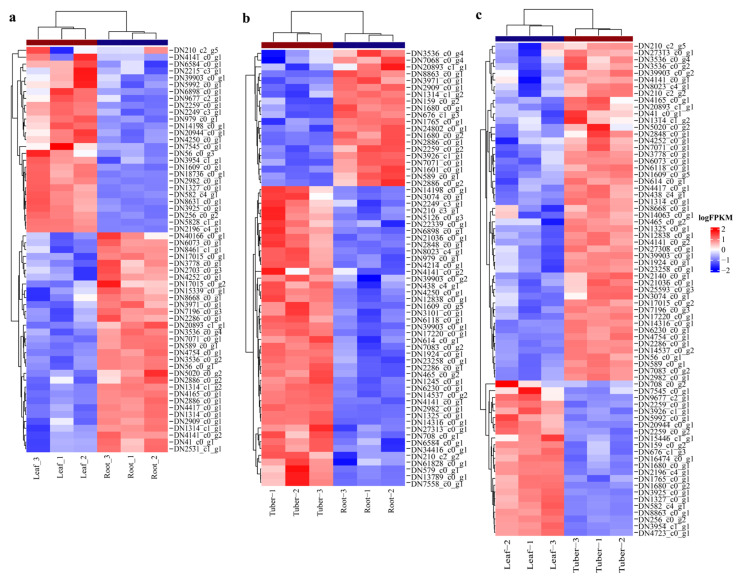
Heatmap clustering analysis of DEGs: (**a**) heatmap clustering analysis of DEGs in Leaf vs. Root; (**b**) heatmap clustering analysis of DEGs in Tuber vs. Root; (**c**) heatmap clustering analysis of DEGs in Tuber vs. Leaf.

To investigate the roles of polysaccharide biosynthesis in tubers, leaves, and roots of *B. striata*, we identified critical genes that are responsible for the high yield of polysaccharides. *B. striata* polysaccharides are primarily composed of glucose and mannose. Hence, the metabolic pathways of fructose and mannose metabolism (ko00051) and starch and sucrose metabolism (ko00500) have been identified as crucial for BSP biosynthesis. Heatmap analysis of DEGs involved in the two pathways revealed the differential expression of these genes when comparing different organs (Figure 6). Specifically, in the Leaf vs. Root comparison, a total of 59 differentially expressed genes were identified, comprising 27 up-regulated and 32 down-regulated genes (Figure 6a). In the Tuber vs. Root comparison, 44 genes were found to be up-regulated, whereas 20 were down-regulated (Figure 6b). The most significant differential expression was observed in the Tuber vs. Leaf comparison, with 50 up-regulated and 23 down-regulated genes (Figure 6c).

**Figure 7:**
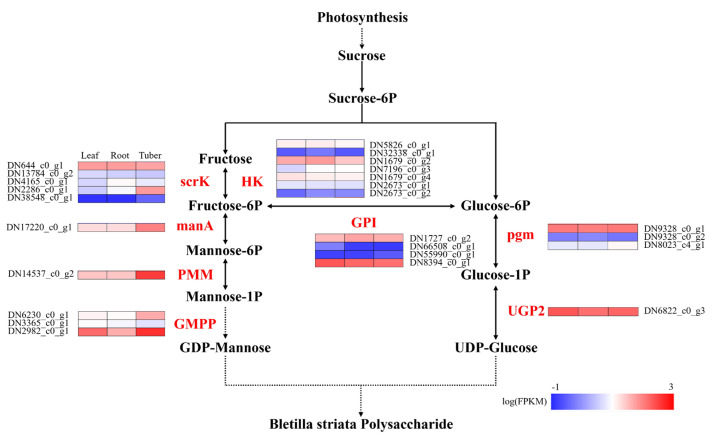
Enzyme gene expression of polysaccharide biosynthesis in different organs of *B. striata*. The black names represent intermediate products in the polysaccharide synthesis pathway, the red names represent key enzymes in the polysaccharide synthesis pathway. Leaf, root, and tuber represent corresponding samples, the numbers on the left represent corresponding enzyme gene IDs, and the blue to red colors represent gene expression levels from low to high.

There are a series of enzymes involved in the biosynthesis pathway of BSP, including hexokinase (HK), fructokinase (scrK), mannose-6-phosphate isomerase (manA), phosphomannomutase (PMM), mannose-1-phosphate guanylyltransferase (GMPP), glucose-6-phosphate isomerase (GPI), phosphoglucomutase (pgm), and UTP-glucose-1-phosphate uridylyltransferase (UGP2). Comparative expression analysis revealed that enzyme genes exhibited significant organ specificity in the synthesis pathway of polysaccharides (Figure 7). Specifically, in the polysaccharide biosynthesis pathway, sucrose is the precursor of polysaccharide synthesis, which is decomposed into fructose and further converted to glucose-6-phosphate. The enzymes scrK and HK play crucial roles in the conversion of fructose to fructose-6-phosphate. The gene DN644_c0_g1, which encoding scrK, exhibits similar expression levels across roots, tubers, and leaves. In contrast, the gene DN1679_c0_g2, encoding HK, shows higher expression levels in roots compared to tubers and leaves. The enzymes such as manA, PMM, and GMPP are responsible for converting fructose-6-phosphate to GDP-mannose. The enzyme genes, including DN17220_c0_g1 encoding manA, DN14537_c0_g2 encoding PMM, and DN6230_c0_g1 and DN2982_c0_g1 encoding GMPP, showed high expression levels in tubers. On the other hand, pgm and UGP2 enzymes catalyze glucose-6-phosphate to UDP-glucose. We identified two genes, DN9328_c0_g1 encoding pgm and DN6822_c0_g3 encoding UGP2, both exhibited high expression levels in all three organs, with particularly high levels in the tubers. GDP monosaccharides and UDP monosaccharides serve as the two key substrates for polysaccharide synthesis, which are catalyzed by glycosyltransferases (GTs); hence, the amount and coordination of their production are crucial for the synthesis of polysaccharides. Notably, DN1727_c0_g2 and DN8394_c0_g1, both encoding GPI, play a crucial coordinating role in balancing the synthesis of GDP monosaccharides and UDP monosaccharides by linking their respective pathways. Similarly, the enzyme genes with high expression levels were found in the roots, tubers, and leaves, with especially high expression in the tubers, indicating that all three tissues can synthesize polysaccharides, but the tuber may be the main storage organ.

### 3.5. RT-qPCR Analysis of Genes Involved in Polysaccharides Biosynthesis

**Figure 8:**
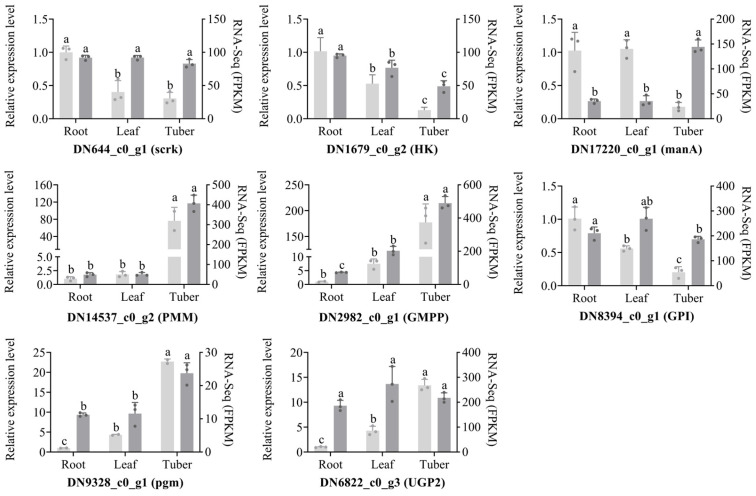
qRT-PCR analysis of eight polysaccharide synthesis genes in different organs of *B. striata*. The vertical axis scale on the left corresponds to the relative mRNA expression, and the vertical axis scale on the right corresponds to the FPKM. Different lowercase letters in the figure represent significant differences, e.g., “a” and “b”.

To validate the organ-specific expression patterns of polysaccharide biosynthesis genes in *B. striata*, we performed RT-qPCR analysis on selected genes. Comparative analysis between transcriptomic data and RT-qPCR results demonstrated high consistency in expression patterns, with the exception of DN17220_c0_g1 (encoding manA) and DN6822_c0_g3 (encoding UGP2) (Figure 8). The polysaccharide synthesis genes DN14537_c0_g2 (encoding PMM), DN2982_c0_g1 (encoding GMPP), DN8394_c0_g1 (encoding pgm), and DN6822_c0_g3 (encoding UGP2) displayed a consistent gene expression pattern in different organs, with high expression in tubers, followed by leaves, and lowest expression in roots. On the other hand, DN644_c0_g1 (encoding scrK) and DN1679_c0_g2 (encoding HK) exhibited the opposite gene expression pattern; DN644_c0_g1 has similar expression levels in the three organs, while DN1679_c0_g2 is highly expressed in the roots and lowest in the tubers. Notably, the expression patterns of DN644_c0_g1 (encoding scrK) and DN8394_c0_g1 (encoding GPI) were similar, showing similar expression levels in the three organs. These findings collectively demonstrate significant organ-specific expression patterns of polysaccharide biosynthesis genes in *B. striata*.

## 4. Discussion

As a traditional Chinese medicinal plant, *B. striata* has been widely used in medical research, particularly for its tubers [3]. Transcriptomics serves as a crucial tool for studying medicinal plants. Identifying differentially expressed genes and elucidating the metabolic pathways of active components provides essential support for the investigation of bioactive constituents in these plants [31]. Huang performed transcriptomic analysis on wild *B. striata* tubers of different growth ages and identified 11,928 DEGs, identified pathways related to growth and development, such as starch and sucrose metabolism and plant hormone signal transduction [32]. Li identified 38 calmodulin-like protein (CML) genes from *B. striata* through transcriptome sequencing, which exhibited tissue specificity. It was found that the differential expression of *BsCML* genes was closely related to the accumulation of metabolites [33]. In this study, we conducted *de novo* transcriptome sequencing to investigate gene expression patterns in roots, tubers of *B. striata*, with a specific focus on uncovering the molecular mechanisms underlying polysaccharide biosynthesis. In comparison to previously published databases [34], this study conducted an in-depth analysis of the differences in the distribution of polysaccharide metabolism pathways, and the expression patterns of key enzyme genes were compared among the tubers, roots, and leaves of *B. striata*. In order to further understand the roles of different organs in polysaccharide biosynthesis in *B. striata*, we investigated the DEGs of roots, tubers, and leaves. The results showed that more DEGs were discovered between the leaf and the other tissues (the tuber and the root), implying that tissues with similar morphology might have more similar molecular bases. The GO enrichment analysis results showed that DEGs were significantly enriched in terms of photosynthesis, plastid, and oxidoreductase activity in leaves, while DEGs were significantly enriched in terms of cell anatomical entities and membrane components in roots and tubers. The KEGG enrichment analysis results revealed that there were significant differences in the enrichment of photosynthesis, starch and sucrose metabolism, glycolysis/gluconeogenesis, fructose and mannose metabolism pathways between leaves and tubers. Simultaneously, there were also significant differences in starch and sucrose metabolism pathways between tubers and roots, indicating significant differences in the distribution of polysaccharide synthesis pathways in the three organs.

BSP is the main active ingredient in *B. striata*, which consists of glucose and mannose and has antioxidant, anti-inflammatory, anti-tumor, and immune-modulatory functions [3,4]. The biosynthetic pathway of BSP is similar to that of *Ginseng* [35], *Polygonatum cyrtonema* Hua [36], and *Dendrobium moniliforme* [37]. It is hypothesized that BSP synthesis primarily occurs in tubers, or that BSP is synthesized in various organs of *B. striata* and then transported to tubers [22]. To test this hypothesis, it is necessary to collect the organs at different growth stages. Transcriptome analysis of early-stage *B. striata* plants allowed us to identify twenty-five key enzyme genes involved in the biosynthesis of BSP. The relatively higher expression of enzyme genes in the tuber may lead to the synthesis of more polysaccharides, consistent with the previous study [31].

Scrk and HK serve as crucial upstream enzymes in the polysaccharide biosynthesis pathway of *B. striata*, catalyzing the conversion of fructose to fructose-6-phosphate. While previous studies have reported a tissue-specific expression pattern of stem > leaf > root for these enzymes in *B. striata* [34], in current study, we found that the expression of *scrk* genes is similar in the three organs, with the highest expression of the *HK* gene in roots and the lowest expression in tubers in *B. striata*. This difference is due to the positive correlation between polysaccharide content and the *scrk* gene, while it is negatively correlated with the *HK* gene [38]. Within the polysaccharide biosynthesis pathway, PMM and GMPP are key enzymes for mannose metabolism, while pgm and UGP2 are essential for glucose metabolism. Collectively, we found that the genes encoding PMM, GMPP, pgm, and UGP2 all exhibited their highest expression levels in the tubers of *B. striata*. The functional importance of these enzymes in polysaccharide biosynthesis is further supported by studies in other species. For instance, *PMM* gene overexpression in *Ganoderma lucidum* significantly increased the polysaccharide yield, which was 1.41 times higher than that of the wild-type strain [39]. Similarly, overexpression of *pgm* gene in *G. lucidum* not only up-regulated the transcript levels of *pgm*, *UGP*, and *GLS* genes but also increased intracellular and extracellular polysaccharides by 40.5% and 44.3%, respectively [40]. Furthermore, overexpression of the *UGP* gene from *Larix Gmelinii* in *Arabidopsis* has been shown to promote vegetative growth and cellulose biosynthesis [41]. These findings collectively highlight the crucial role of these enzymatic genes in polysaccharide metabolism, and their comprehensive analysis will provide valuable insights into the molecular mechanisms of polysaccharide biosynthesis in medicinal species.

## 5. Conclusions

As an important medicinal substance, BSP has various beneficial functions. In this study, transcriptome data in different organs of *B. striata* were analyzed. Our results revealed the organ-specific differential expression of BSP biosynthesis-related genes and identified key enzyme genes. These results provide a reference for the accumulation mechanism of polysaccharides and a basis for the rational utilization of *B. striata*.

## Data Availability

The data that support the findings of this study are available from the corresponding author upon reasonable request.

## Supplementary Materials

The following supporting information can be downloaded at: https://www.mdpi.com/article/10.3390/genes16050558/s1, Table S1: Primer information of RT-qPCR; Table S2: Sequencing data statistics; Table S3. Distribution of transcripts different lengths; Table S4. Unigene functions annotation.

## Abbreviations

The following abbreviations are used in this manuscript:

BSP	*Bletilla striata* polysaccharide
NR	Non-Redundant Protein Sequence Database
GO	Gene Ontology
KEGG	Kyoto Encyclopedia of Genes and Genomes
KOG	Eukaryotic Orthologous Groups
Swiss-Prot	SwissProt Database
DEGs	Differentially Expressed Genes
RT-qPCR	Quantitative Reverse Transcriptase Polymerase Chain Reaction
SARS-CoV-2	Severe Acute Respiratory Syndrome Coronavirus 2
SWEET	sugars will eventually be exported transporter
sacA	beta-fructofuranosidase
4-CL	4-coumarate-CoA ligase
GLS	glutaminase gene
scrK	fructokinase
HK	hexokinase
manA	mannose-6-phosphate isomerase
PMM	phosphomannomutase
GMPP	mannose-1-phosphate guanylyltransferase
GPI	glucose-6-phosphate isomerase
pgm	phosphoglucomutase
UGP2	UTP-glucose-1-phosphate uridylyltransferase
GTs	glycosyltransferases
