# Supplementary material for: De Novo Transcriptome Sequencing Analysis Revealed the Expression Patterns of Genes in Different Organs and the Molecular Basis of Polysaccharide Synthesis in Bletilla striata
Source: Genes (Basel). 2025 May 6;16(5):558. doi: 10.3390/genes16050558 (PMC12111575; doi:10.3390/genes16050558)
Supplement: Supplementary file 1 [file genes-16-00558-s001.zip › Supplementary Materials.pdf]

Table S1. Primer information of qRT-PCR.

Genes	Primer sequence (5'-3')
scrK	F: CCTGACCGGCAAAGATTCGG
	R: CAAATGCTTCTCCAGCGCCC
HK	F: TCTCAGAGTAGTCGGGAGCA
	R: TCAGTGGCAACATCCCTTCC
manA	F: CCGGATAAGGAGTTGGCTGG
	R: AGCTCCCAAATCTCAGGCAC
PMM	F: ACAAGGGCGGAAATGACTTTG
	R: TTGCTTTGCCAGGAAGTGGA
GMPP	F: ATCGTCGGCCAAACTTGCTT
	R: TCCGCGCATCACAGTACATC
GPI	F: ACCTGGAACAAATGGTCAGCA
	R: GCCCTGATTACACTCGGAGG
pgm	F: GGCTTCTTTCACACCGTCTAC
	R: GAACCAGTAGCCCGTCCTTG
UGP2	F: ATCTGGTTTCATCAGCCTTGTCT
	R: ATGGCACGACAACCTCATCG
GADPH	F: GCTAAAGACGCTGTCACTGAG
	R: GAGACCAGAAACTTACCCTCG

Table S2. Sequencing data statistics.

Sample	Clean Reads	Clean base(bp)	GC (%)	Q20 (%)	Q30 (%)
Leaf_1	21,678,883	6,503,664,900	44.23	99.24	97.52
Leaf_2	21,130,006	6,339,001,800	44.37	98.86	96.58
Leaf_3	20,722,284	6,216,685,200	44.73	99.08	97.02
Root_1	20,201,786	6,060,535,800	43.89	99.15	97.26
Root_2	21,250,939	6,375,281,700	43.84	97.33	93.93
Root_3	19,610,668	5,883,200,400	44.50	99.20	97.41
Tuber_1	19,731,654	5,919,496,200	45.85	99.14	97.20
Tuber_2	20,670,485	6,201,145,500	46.05	99.12	97.14
Tuber_3	21,608,753	6,482,625,900	45.86	98.87	96.63

Note: Leaf: leaf of *B. striata*; Root: root of *B. striata*; Tuber: tuber of *B. striata*; 1-3: The sample repeats; Q20: Percentage of bases with clean reads' mass value greater than or equal to 20; Q30: Percentage of bases with clean reads' mass value greater than or equal to 30.

Table S3. Distribution of transcripts different lengths.

Length	Number	Percent (%)
Total	175,440	100.00
<500	66,031	37.64
500~1000	35,588	20.28
1000~2000	36,767	20.96
>2 000	37,054	21.12

Table S4. Unigene functions annotation.

Database	Annotated	Percent (%)
All [1]	96,418	100.00%
NR [2]	38,542	39.97%
GO [3]	30,554	31.69%
KEGG [4]	12,848	13.33%
KOG [5]	5921	6.141%
Swiss-Prot [6]	57,530	59.67%

[1] All: Sum of the above 5 databases; [2] NR: Non-Redundant Protein Sequence Database; [3] GO: Gene Ontology; [4] KEGG: Kyoto Encyclopedia of Genes and Genomes; [5] KOG: Eukaryotic Orthologous Groups; [6] Swiss-Prot: SwissProt Database.
